# An Extensive Tail Sign in Primary Thoracic Myxofibrosarcoma: A Case Report

**DOI:** 10.70352/scrj.cr.26-0328

**Published:** 2026-10-07

**Authors:** Megumi Kobayashi, Ei Miyamoto, Yuki Morimura, Yuki Ohsumi, Takeshi Kubo, Tatsuo Nakagawa

**Affiliations:** 1 Department of Thoracic Surgery, Tenri Hospital, Tenri, Nara, Japan; 2 Department of Radiology, Tenri Hospital, Tenri, Nara, Japan

**Keywords:** myxofibrosarcoma, tail sign, chest wall tumor

## Abstract

**INTRODUCTION:**

Myxofibrosarcoma is a malignant soft tissue tumor of fibroblastic origin, occurring mainly in the extremities but extremely rarely in the thoracic trunk. Myxofibrosarcoma typically appears as a slow-growing, painless mass but features a frequent local recurrence due to microscopic extension beyond visible margins. On MRI, the “tail sign”—which indicates subtle spread along the fascial plane—is known to be a potential risk factor for recurrence.

**CASE PRESENTATION:**

The patient was a 73-year-old woman complaining of continuous right back pain. Chest CT showed a poorly contrast-enhanced mass involving the 5th and 6th right ribs, as well as a separate nodule. The biopsy confirmed a diagnosis of myxofibrosarcoma. The mass exhibited hypointensity on T1-weighted images and hyperintensity on T2-weighted images on MRI; on contrast-enhanced T1-weighted images and STIR images, a tail sign extending toward a small satellite nodule was observed. The patient underwent an extended resection involving the 5th through 7th ribs. A total of 61.2 Gy radiation was irradiated to the paravertebral region neighboring the tumor where the surgical margin was poor. A local recurrence was detected in the anterolateral region of the tumor resection site 5 months after surgery. The patient was treated with sequential chemotherapy using doxorubicin, eribulin, and pazopanib and is alive at 26 months after surgery without distant metastasis.

**CONCLUSIONS:**

The current case exemplifies the aggressive local behavior of thoracic myxofibrosarcoma, especially when a long tail sign appears.

## Abbreviations

FNCLCC
: Fédération Nationale des Centres de Lutte Contre le Cancer
STIR
: short tau inversion recovery

## INTRODUCTION

Myxofibrosarcoma is a connective tissue neoplasm of fibroblastic origin set in a myxoid matrix,^1)^ and has been considered as a myxoid variant of malignant fibrous histiocytoma.^2)^ Myxofibrosarcoma is one of the most common soft tissue sarcomas in the elderly. It arises mainly in the extremities and rarely in the thoracic trunk.^1,2)^ Myxofibrosarcoma is known for its locally aggressive behavior. The 5-year recurrence-free survival rate ranges from 25% to 82%.^2–4)^ A tail sign often appears on MRI as a continuous high signal intensity extending from the tumor, representing tumor cell infiltration along the fascial plane.^5,6)^ We herein report a case of an extremely rare thoracic myxofibrosarcoma with a long tail sign.

## CASE PRESENTATION

A 73-year-old woman was referred to our hospital with a complaint of pain in her right chest and back. Chest CT revealed a 7.5 × 4.5 × 4.8-cm hypovascular mass causing destruction of the right 6th rib (**Fig. 1A**) and a 1.3-cm satellite nodule at the anterior 4th intercostal space (**Fig. 1B**). On MRI, the primary tumor exhibited low signal intensity on T1-weighted images and high signal intensity on T2-weighted images. Continuous high-intensity signals extending from the primary tumor toward the satellite lesion and the surrounding tissue along both the external and internal surfaces of the intercostal fascia and latissimus dorsi fascia, referred to as the tail signs in multiple layers, were identified on contrast-enhanced T1-weighted (**Fig. 1C**) and STIR images (**Fig. 1D**). Significant uptake of fluorodeoxyglucose was identified at the main lesion with a maximum standardized uptake value of 4.8 on fluorodeoxyglucose-PET, and mild uptake in the satellite lesion. No other nodules or distant metastatic lesions were identified. CT-guided needle biopsy suggested a diagnosis of myxofibrosarcoma or dermatofibrosarcoma protuberans.

**Fig. 1:**
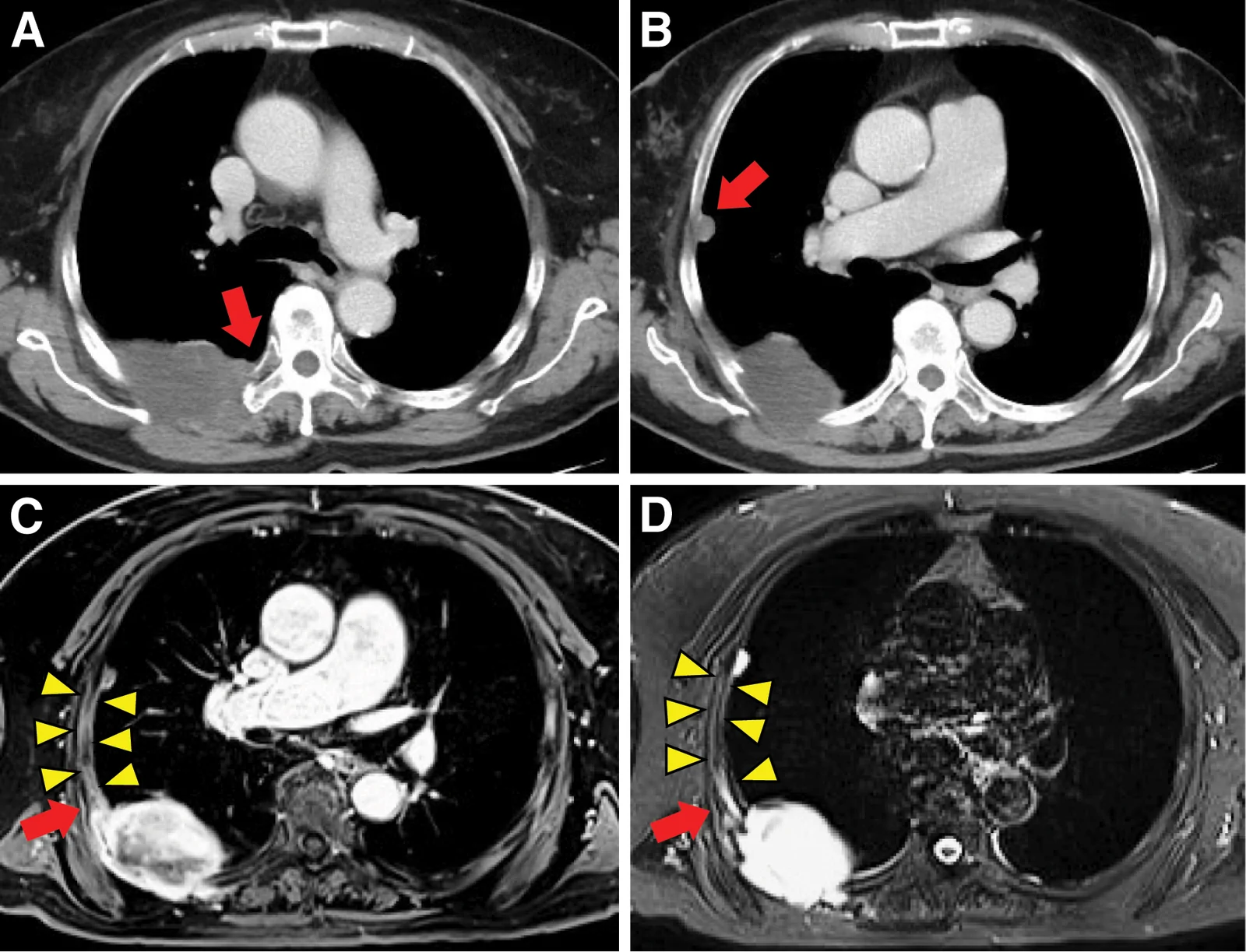
CT images of the chest (**A** and **B**) and axial MRI (**C** and **D**). (**A**) The hypovascular mass was located adjacent to the costovertebral joint of the 6th rib (red arrow). (**B**) A satellite nodule (red arrow) was observed in the 4th intercostal space. (**C**) Contrast-enhanced fat-suppressed T1-weighted image demonstrates high signal intensity extending from the mass toward the small satellite nodule. The arrowheads indicate thickened intercostal fascia and periosteum; the red arrow indicates thickened latissimus dorsi fascia. (**D**) STIR image reveals comparable features. STIR, short tau inversion recovery

The surgery was performed via a right posterolateral thoracotomy in the prone position through a 25-cm skin incision. During manipulation of the lateral chest wall, the operating table was tilted to the left. The tumor had infiltrated the chest wall, including the ribs and the serratus anterior muscle. The latissimus dorsi muscle was preserved, and the thoracic cavity was initially entered through the 7th intercostal space in the lateral chest wall. The distal portions of the 5th to 7th ribs were resected first. After partial resection of the erector spinae muscle, the trapezius muscle was split along its fibers at the posterior midline, allowing the orthopedic surgeons to transect the transverse processes of the 5th and 6th thoracic vertebrae. The proximal ends of the 5th to 7th ribs were then separated from the vertebrae at the costovertebral joints. The resection margin was grossly minimal at the proximal aspect of the 6th rib (**Fig. 2A** and **2B**). The satellite nodule was resected separately from the main tumor. Chest wall reconstruction was performed using a 2-mm-thick, 150 × 200-mm Gore-Tex sheet sutured to the chest wall from the intrathoracic side. The operative time was 211 minutes, and the estimated blood loss was 210 mL. No blood transfusion was required.

**Fig. 2:**
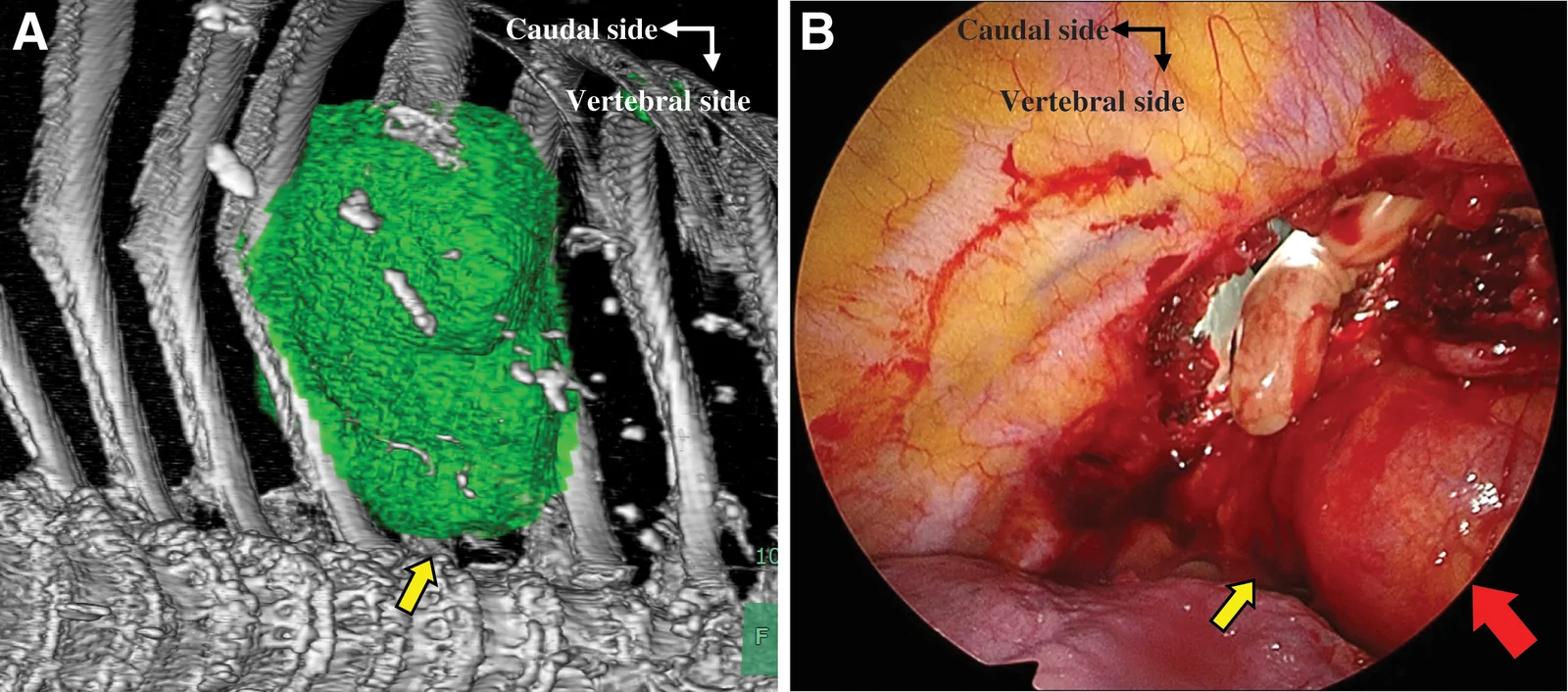
(**A**) 3D CT image. (**B**) Intraoperative video image. The red arrow indicates the tumor. The yellow arrow indicates the proximal end of the 6th rib, where the surgical margin was grossly minimal.

The tumor was a soft, yellowish-white mass measuring 8.4 cm in diameter and centered on the 6th rib, which was extensively destroyed within the tumor (**Fig. 3**). Gross examination showed that the surgical margin was narrowest at the proximal end of the 6th rib. The margin around the satellite nodule was greater than 1 cm (**Fig. 4**). Pathological examination confirmed that both the proximal and distal ends of the resected specimen were tumor-negative. The encapsulated tumor was exposed on the pleural surface near the proximal end of the resected 6th rib (**Fig. 3**, yellow arrow), where the surgical margin was considered minimal. Hematoxylin and eosin staining showed irregular proliferation of short spindle and stellate tumor cells in a myxoid stroma, with nuclear pleomorphism (**Fig. 5A**). Immunohistochemically, the tumor was mostly positive for CD34 (**Fig. 5B**), focally positive for α-smooth muscle actin (**Fig. 5C**), and negative for ALK, desmin, and S-100. These findings were consistent with myxofibrosarcoma (pT2NxM0, FNCLCC grade 1).

**Fig. 3:**
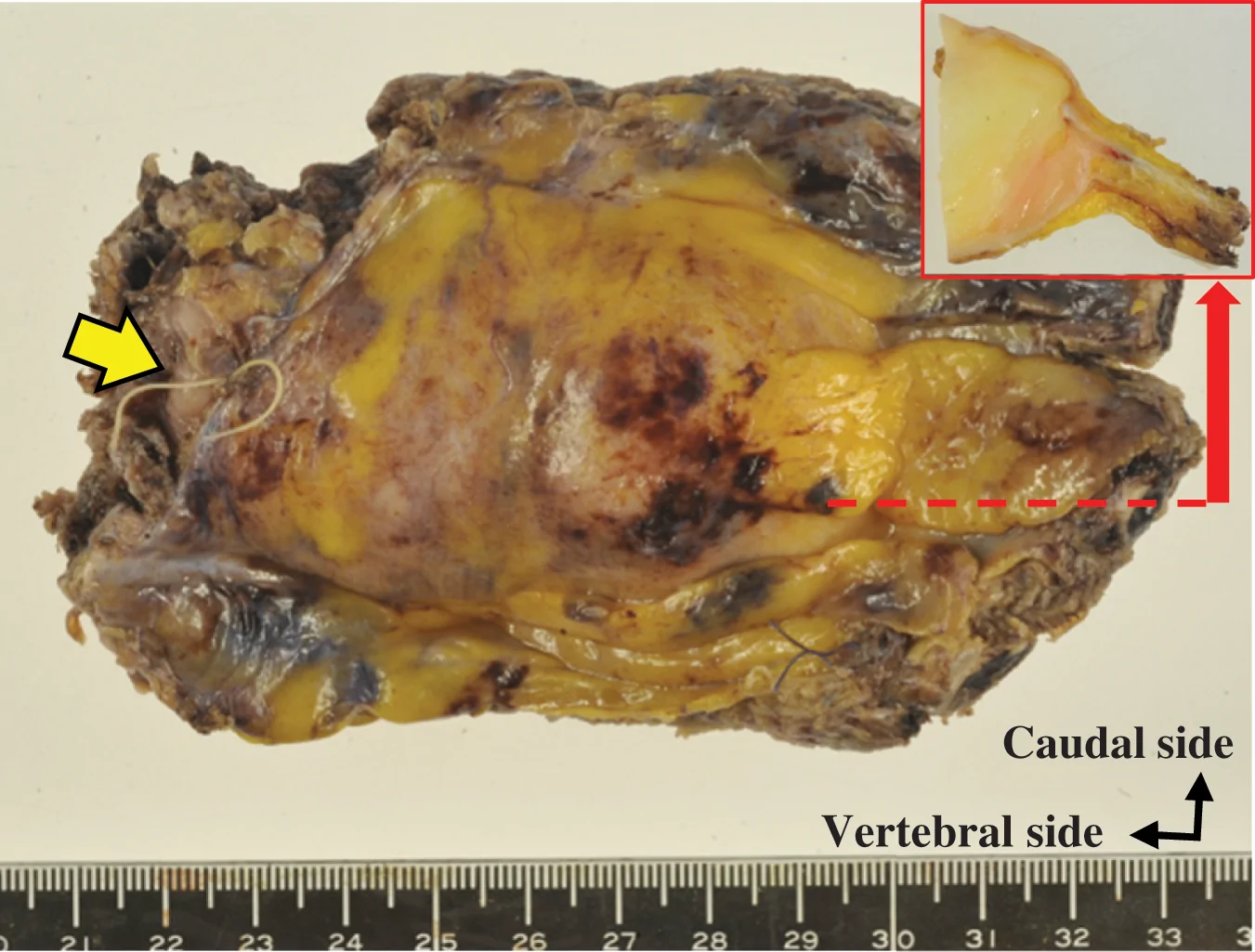
Macroscopic pathological image of the main tumor. Yellowish-white soft tumor. The pleural side is shown anteriorly, and the caudal and vertebral sides are indicated by arrows. The yellow arrow indicates the encapsulated tumor exposed on the pleural surface, where the surgical margin was expected to be closest. The dashed line indicates the cut surface. The minor panel shows a cross-sectional view of the tumor along with the rib. The 6th rib was almost completely resorbed within the tumor.

**Fig. 4:**
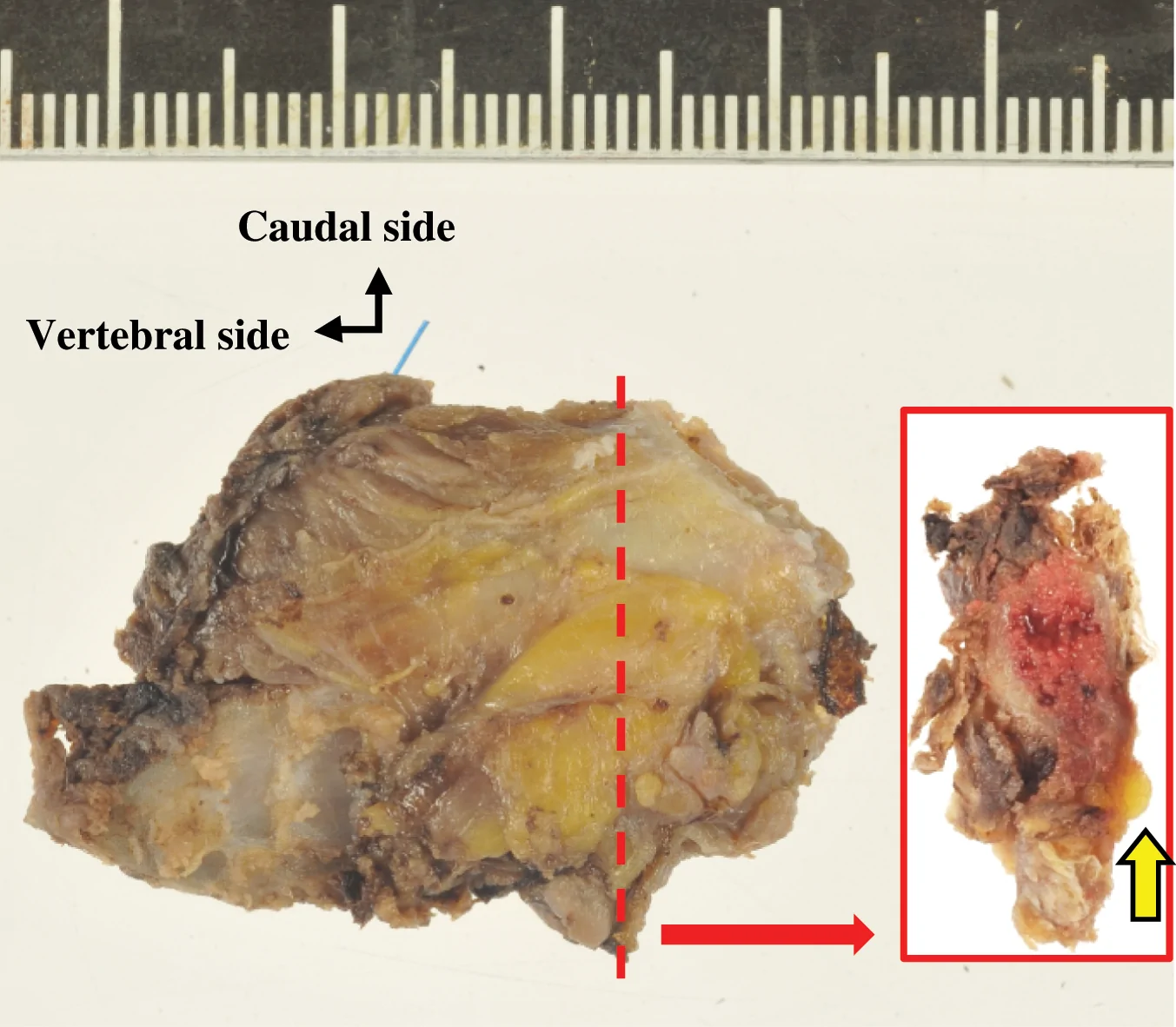
Gross pathological findings of the satellite nodule. The pleural side is shown anteriorly. The dashed line indicates the cut surface. The yellow arrow in cross-sectional view of the specimen indicates the satellite nodule.

**Fig. 5:**
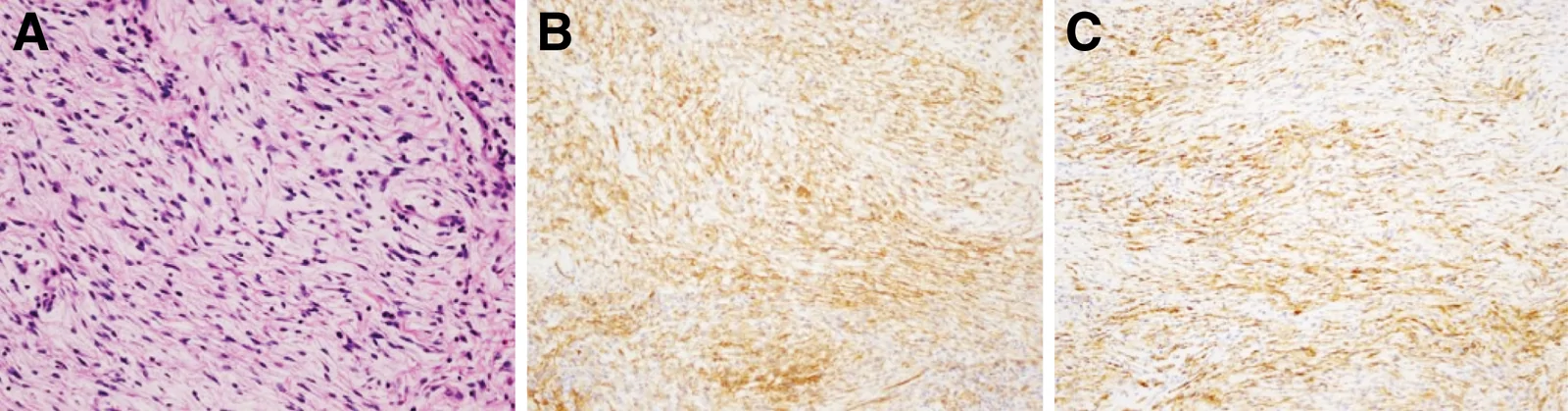
Histopathological findings of the resected specimen: (**A**) ×200, (**B**, **C**) ×100. (**A**) Hematoxylin and eosin staining showing short spindle cells within a myxoid stroma. Immunohistochemically, the tumor was (**B**) diffusely positive for CD34 and (**C**) focally positive for α-smooth muscle actin.

Postoperative radiotherapy (61.2 Gy) was administered against the paravertebral region where the resection margin was minimal. A local recurrence occurred 5 months after surgery (**Fig. 6**), and systemic chemotherapy (doxorubicin, eribulin, pazopanib) was administered. At 26 months post-surgery, there was no evidence of distant recurrence.

**Fig. 6:**
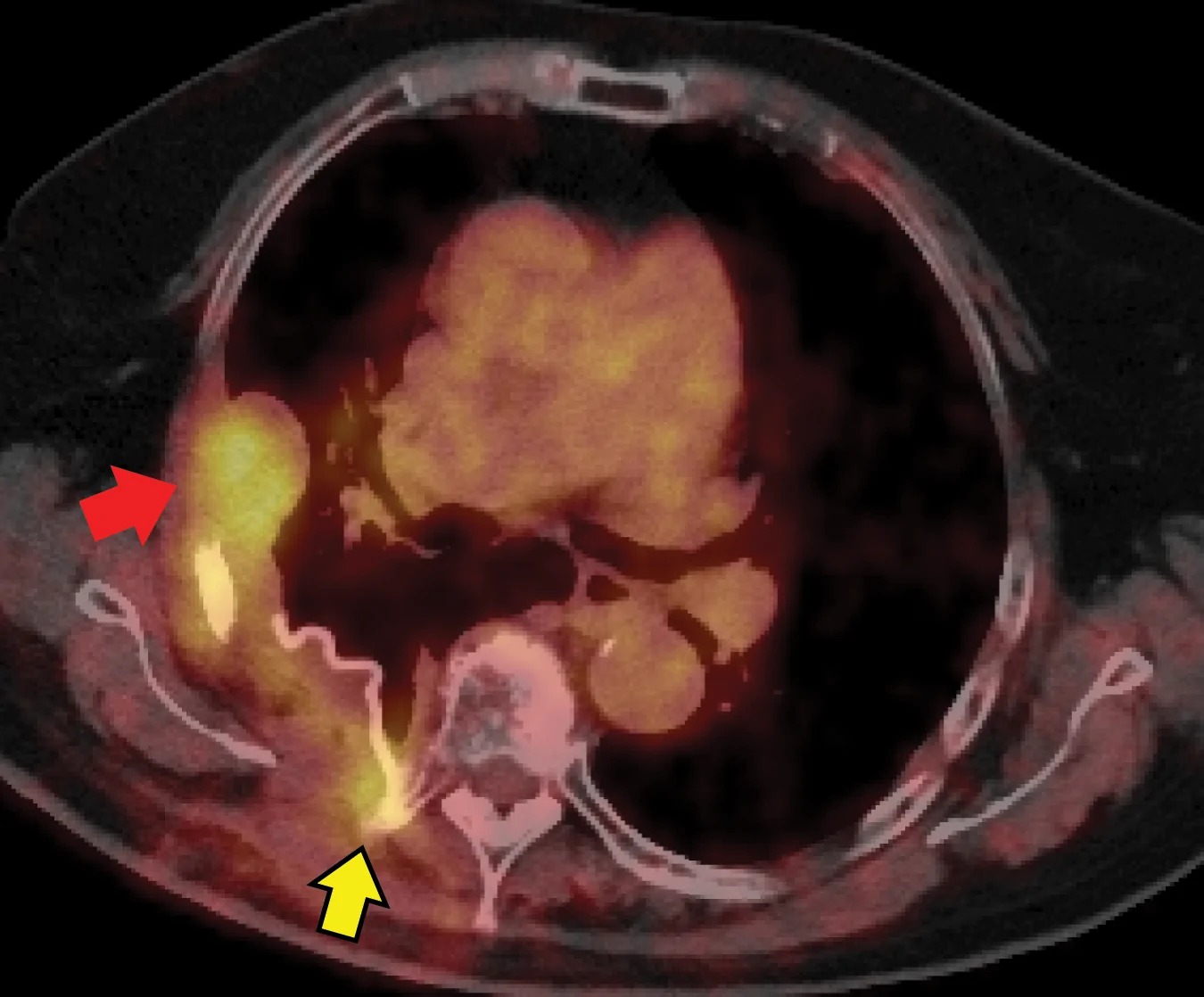
Postoperative imaging obtained 5 months after surgery. Fluorodeoxyglucose PET/CT shows intense fluorodeoxyglucose uptake in the recurrent nodule in the 3rd intercostal space (red arrow) and in the paravertebral area (yellow arrow).

## DISCUSSION

Myxofibrosarcoma of the thoracic wall is extremely rare.^1)^ Myxofibrosarcoma accounts for approximately 5% of soft tissue sarcomas,^2)^ and mainly affects elderly patients.^2,6)^ It most commonly arises in the dermal and subcutaneous tissues of the lower extremities (48%–60%), followed by the upper extremities (24%–34%), trunk (12%–15%), retroperitoneum or mediastinum (8%), and head and neck region (1%–3%).^2,7,8)^ Although myxofibrosarcoma is typically a slow-growing, partially nodular, and relatively painless tumor,^2,9)^ the patient in this case had severe back pain caused by rib invasion, suggesting locally aggressive tumor growth. The FNCLCC grading system is widely used for soft tissue sarcomas and classifies tumors into 3 grades based on mitotic activity, necrosis, and differentiation.^10,11)^ In myxofibrosarcoma, a higher FNCLCC grade has been associated with an increased risk of distant metastasis,^6,7)^ but not with local recurrence.^3,7,10,12)^ Consistent with these reports, this FNCLCC grade 1 myxofibrosarcoma showed no distant metastasis during 26 months of follow-up, despite its locally aggressive behavior.

To the best of our knowledge, the present case is the first to specifically document the extensive tail sign in primary thoracic myxofibrosarcoma. On MRI, myxofibrosarcoma presents with low signal intensity on T1-weighted images, lower than that of normal muscle, and variable high signal intensity on water-sensitive sequences, depending on the myxoid matrix content.^5,6)^ The tail sign can be detected as a curvilinear extension of the tumor along fascial planes on T2-weighted images with fat suppression and STIR or as thickened, enhancing fascia on T1-weighted images with fat suppression after gadolinium administration.^6,13)^ Recent studies have suggested that the tail sign is associated with an increased risk of postoperative local recurrence,^14)^ although its relationship with overall survival remains unclear. In local tumor spread, the deep peripheral fascia forms the anatomical boundary of the subcutaneous compartment and can act as a barrier to trans-fascial extension.^5)^ The tail sign is thought to reflect microscopic tumor infiltration into subcutaneous tissue and fascia, sometimes with satellite tumor cells.^6,13)^ This discontinuous, skip-like extension of myxofibrosarcoma can make pathological margin assessment vulnerable to sampling error^15)^ and may explain frequent local recurrence even after apparently margin-free resection.^3,7)^ In the present case, no tumor cells were identified at the outer edge of the resected tissue; however, infiltrative residual components corresponding to the tail signs in multiple layers may have contributed to early local recurrence.

Regarding the treatment for myxofibrosarcoma, radical tumor resection with achieving clear surgical margins is critically important.^12)^ In the present case, the operation was performed in the complete-to-tilted prone position, which provided a favorable surgical field with access to the lateral chest wall for the thoracic surgeons and to the costovertebral joints for the orthopedic surgeons. Since the tail sign is particularly difficult to identify intraoperatively, achieving negative margins while taking the tail signs in multiple layers into account is deemed as especially challenging. Although the radical resection of the main tumor, including the satellite nodule and the tail sign extending toward it was achieved, we may not have fully appreciated the clinical significance of the additional tail signs extending into the surrounding tissues in multiple layers. If a method had been available to clearly visualize these multiple tail signs during surgery, recurrence-free surgical treatment might have been achieved. To secure an optimal surgical margin in cases with infiltrative extension into the surrounding tissues, the potential role of near-infrared-guided intraoperative navigation in achieving negative surgical margins in soft tissue sarcoma is being investigated.^15–17)^

## CONCLUSIONS

We report a rare case of thoracic myxofibrosarcoma that underscores the clinical relevance of the tail sign on preoperative imaging. Identification of features suggestive of locally aggressive behavior, such as the tail sign on MRI, is important for treatment decisions on myxofibrosarcomas.

## DECLARATIONS

### Availability of data and materials

The datasets supporting the conclusions of this article are included within the article.
